# Over-expression of miR-34c leads to early-life visceral fat accumulation and insulin resistance

**DOI:** 10.1038/s41598-019-50191-3

**Published:** 2019-09-25

**Authors:** Philip H. Jones, Brian Deng, Jessica Winkler, Arin L. Zirnheld, Sarah Ehringer, Vikranth Shetty, Matthew Cox, Huy Nguyen, Wen-Jun Shen, Ting-Ting Huang, Eugenia Wang

**Affiliations:** 1 Advanced Genomic Technology, LLC, Louisville, KY USA; 2 Palo Alto Veterans Institute for Research, Palo Alto, CA USA; 3 Department of Neurology and Neurological Sciences, Stanford University School of Medicine, Stanford, CA USA; 4 Department of Endocrinology, Stanford University School of Medicine, Stanford, CA USA; 5 Geriatric Research, Education, and Clinical Center, VA Palo Alto Health Care System, Palo Alto, CA USA

**Keywords:** Pre-diabetes, Homeostasis, Obesity

## Abstract

Overweight children and adolescents are at high risk for adult and late life obesity. This report investigates some underlying mechanisms contributing to obesity during early life in an animal model. We generated a strain of transgenic mice, cU2, overexpressing human microRNA 34c, a microRNA functionally implicated in adipogenesis. Male and female cU2 mice exhibit significant weight gain, accompanied by marked increase in abdominal fat mass and metabolic abnormalities, including reduction of both glucose clearance rate and insulin sensitivity, as early as two months of age. Adipogenesis derailment at this early age is suggested by decreased expression of adiponectin, the fat mass and obesity-associated gene, and the adiponectin receptor R1, coupled with a reduction of the brown fat biomarker PAT2 and the adipogenesis inhibitor SIRT1. Notably, adiponectin is an important adipokine and an essential regulator of glucose and fatty acid homeostasis. cU2 mice may provide a crucial animal model for investigating the role of miR-34c in early onset insulin resistance and visceral fat mass increase, contributing to accelerated body weight gain and metabolic disorders. Intervention in this dysregulation may open a new preventive strategy to control early-life weight gain and abnormal insulin resistance, and thus prevalent adult and late life obesity.

## Introduction

Available animal models for obesity are numerous, exemplified by the nutritional approach *via* high fat diet regimen, and genetic alteration of leptin signaling leading to overeating^1^. Use of these models has unveiled a wealth of knowledge concerning adipogenic pathways and appetite control hormones *via* hypothalamus^2^. These animal model studies clearly demonstrate that eating habits, in addition to other life style habits such as lack of exercise, are among many factors contributing to adult obesity. Nonetheless, this approach does not address the emerging suggestion of a “developmental origin of obesity”^3–5^ occurring in early life, from prenatal to childhood and adolescent stages. Obesity at these stages is increasingly associated with environmental exposure and parental life style, such as smoking during gestation, maternal obesity, and paternal high fat diet exposure, leading to derailment of adipogenic pathways as an underlying mechanism for early life overweight^6–9^.

Adipogenesis is regulated by several transcription factors and microRNAs (miRNAs) that modulate adipocyte proliferation and differentiation^10^. Among the adipogenesis-associated miRNAs, members of the miRNA-34 (miR-34) cluster, most notably miR-34a, are upregulated during adipogenesis and positively correlated with body mass index^10^. The miR-34 family includes three major members, miR-34a, −34b, and −34c; miR-34a and miR-34b/c are transcribed from two different transcription units. In addition to regulating adipogenic pathways, miR-34 family members are best known as tumor suppressors; they are transcriptionally activated by p53, and can cause cell cycle arrest and suppress the expression of cell survival factors such as Bcl2^11,12^.

Given the connection between miR-34 and p53, early childhood influences on adipogenesis may be manifested as molecular stress impacting on targeted signaling, most notably the miR-34/p53 axis, which can be readily activated by many genotoxic and environmental insults. Paradoxically, activation of miR-34 may reduce SIRT1, another target of miR-34 family members, and suppress brown fat formation and fibroblast growth factor 21 (FGF21) signaling^13^. Moreover, elevated miR-34a also suppresses nicotinamide phosphoribosyl transferase (NAMPT), resulting in reduced NAD + biosynthesis in the liver of obese mice fed a high fat diet^14^. Similarly, miR-34c suppresses SIRT1 expression, and is identified as one of the adipogenesis-regulated miRNAs in 3T3-L1 pre-adipocytes, primary mouse adipocytes, and human adipose tissues^15,16^. Thus, excessive activation of miR-34 cluster expression, while endowing the gain of tumor suppression in early life, may be at the cost of altering normal adipogenic pathways and leading to early life obesity.

Obesity is a recognized risk factor for metabolic syndrome, Alzheimer’s disease, cardiovascular disorders, Type 2 diabetes, and cancer^17–20^. Thus, elevated levels of members of the miR-34 cluster may not only induce obesity, but also create high risks for late life-associated diseases. Interestingly, caloric restriction starting at 3 months of age maintains the expression of miR-34a without increase, as seen in *ad lib* fed counterparts, during aging^21^. Emerging from this scenario is the mystery of whether the “developmental origin of obesity” may be associated with abnormally high miR-34 expression early in life, with abnormal cluster-regulated adipogenic pathways, and whether this molecular dysregulation, continuing into adult and late life, may contribute to adult obesity and high risks for late life, age-dependent disorders.

In the present study, we report generation of the cU2 transgenic mouse strain, which ubiquitously over-expresses human miR-34c (hsa-miR-34c) from birth. The most important phenotypic observation is accelerated weight gain and body fat mass increase, followed by significant abdominal distension. Impaired glucose metabolism is observed very early, with increased insulin resistance evident at one month of age, and obesity-associated dysregulation of adipogenic pathways a few months later. Thus, the overexpression of hsa-miR-34c by molecular manipulation may contribute to three key physiological changes in early life: **a**. increased body fat indices; **b**. dysregulated insulin sensitivity/glucose metabolism; and **c**. altered adipogenic regulation. All three changes pose high risks for obesity later in adult life.

## Results

### Generation and confirmation of cU2 transgenic mice

**Figure 1:**
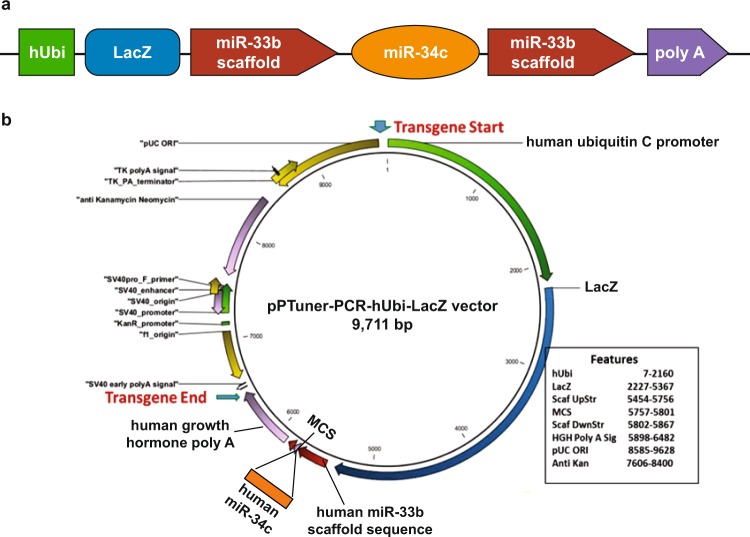
Transgenic construct of human miRNA 34c (hsa-miR-34c). (**a**) Schematic presentation of the transgene construct, with human ubiquitin promoter (hUbi) driving the expression of LacZ reporter and human miR-34c, followed by the human growth hormone polyadenylation sequence. The human miR-34c is flanked by the human miR-33b scaffold sequence. Because human miR-33b is not found in mice, it is used as a unique sequence in the transgene construct to facilitate genotyping of transgenic mice. (**b**) Vector structure of the hsa-miR-34c transgene. The expression vector pPTuner was used for the construction of the miR-34c transgene.

**Figure 2:**
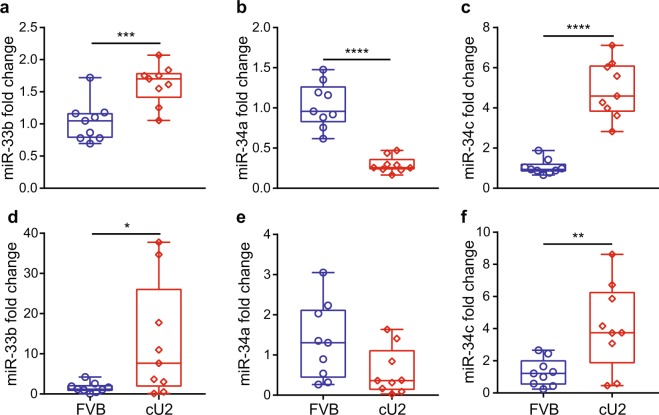
Expression levels of miR-33b, miR-34a, and miR-34c in kidney (**a**–**c**) and liver (**d**–**f**) at 3 months of age. Expression levels were examined by quantitative RT-PCR (RT-qPCR). Box plots are used to show the relative expression levels between FVB and cU2. N = 9 each. Only tissues collected from male mice were examined. *p < 0.05; ***p < 0.001; ****p < 0.0001.

**Figure 3:**
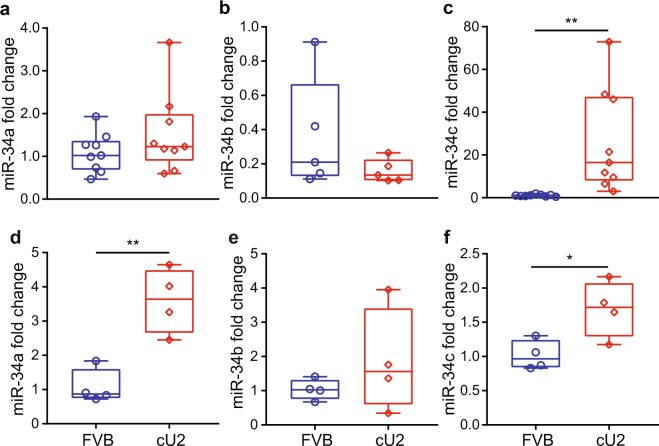
Expression of miR-34a (**a**,**d**), miR-34b (**b**,**e**), and miR-34c (**c**,**f**) in adipose tissues of male (**a**–**c**) and female (**d**–**f**) mice at 3 months of age. Expression levels were examined by RT-qPCR. Box plots are used to show the relative expression levels between FVB and cU2. Males, N = 9 each for miR-34a and miR-34c and n = 5 for miR-34b; females, N = 4 each. *p < 0.05; **p < 0.01.

Several founders were generated by microinjection of the hsa-miR-34c transgene; the line that went germline and remained stable was designated cU2 (Figs 1 and S1). To validate the expression of hsa-miR-34c in the transgenic mice, quantitative PCR (qPCR) assays of miR-34c expression in several tissues were performed (Figs 2 and 3). To verify that the elevated expression of miR-34c in cU2 mice is due to the presence of the hsa-miR-34c transgene, we also evaluated the concordance in the expression levels between miR-34c and hsa-miR-33b in cU2 mice. In addition, levels of miR-34a expression were evaluated for the possible impact of the transgene upon the endogenous miRNA expression of this microRNA cluster. Significant levels of hsa-miR-33b were detected in the kidneys of male cU2 mice (Fig. 2a), which corresponded to high levels of miR-34c expression (Fig. 2c). On the other hand, miR-34a expression did not exhibit the same trend of increase as either miR-34c or miR-33b (Fig. 2b). If any trend was observed at all, kidney tissues of the transgenic mice exhibited reduced levels of miR-34a, compared to FVB controls. Similar patterns of hsa-miR-33b, miR-34a, and miR-34c expression were detected in the livers of cU2 mice (Fig. 2d–f). However, a wider range of inter-animal variations for the transgene expression was observed in the liver (Fig. 2d–f).

### Expression levels of all three members of the miR-34 cluster in visceral fat of both genders

To verify that transgene miR-34c is expressed in visceral fat tissues at equal levels in both genders, RNAs isolated from this tissue of both male and female mice were used for qPCR assays. In addition, we determine the expression levels of sister miRNAs, mmu-miR-34a and −34b, following the same quantification protocol described in Methods. Significantly elevated miR-34c expression was indeed observed in the visceral fat of both male and female cU2 mice (Fig. 3c,f), but no significant difference was observed in mouse miR-34b levels in either gender (Fig. 3b,e). Interestingly, significantly elevated levels of mouse miR-34a expression were observed in the visceral fat of cU2 female mice (Fig. 3d), but were not noted in cU2 males (Fig. 3a). The original data points of all qPCR assays for all panels of Figs 2 and 3 are included in Table S2, with the statistical analysis described in Methods.

### Changes of body fat, glucose tolerance, and insulin sensitivity at young age

**Figure 4:**
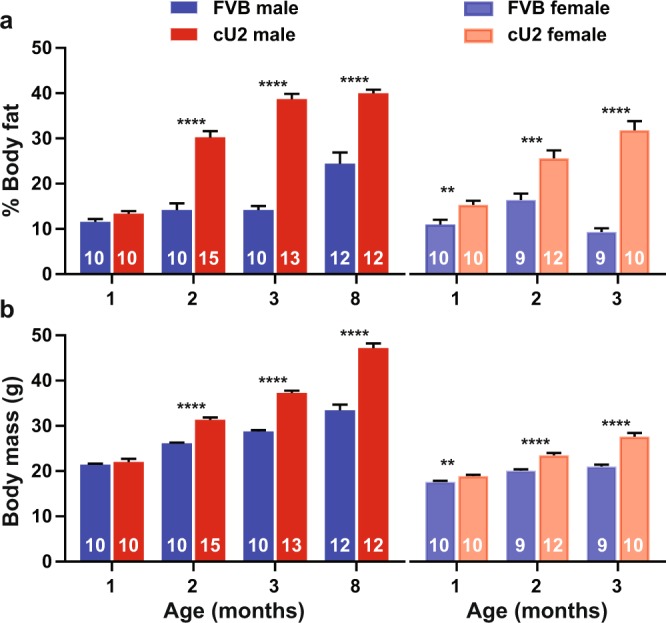
Body composition examined by dual-energy X-ray absorptionometry (DEXA) analysis. (**a**) % body fat, and (**b**) total body mass calculated from DEXA scan. Sample sizes are indicated within the bar graphs. Multiple *t*-tests were used to compare differences between age-matched FVB and cU2 mice. The false discovery rate was set at 1%. All data shown as mean ± SEM. **p < 0.01; ***p < 0.001; ****p < 0.0001.

DEXA measurements of body composition showed a slightly higher percentage of body fat in both genders of cU2 mice at one month of age, but the increase was only significantly different from that of controls in female mice (Fig. 4a). However, by 2 months of age, the difference in body fat content reached significant levels in both genders, and this trend continued to 3 months of age. In the available older male mice, we were able to measure body composition up to 8 months of age, and demonstrated persistently higher levels of body fat in cU2 mice (Fig. 4a).

The elevation of body fat content at 2 months of age corresponded to a significant increase in body mass from global measurement by the DEXA scan (Fig. 4b). Comparison between panels A and B shows an accelerated rate of body fat and body mass increase in cU2 mice, especially between 1 and 3 months of age. During these two months, the average body fat increase in both male and female cU2 mice accounted for 70% and 64% of body mass increase, respectively, whereas the body fat increase in FVB male mice only accounted for 20% of body mass increase during the same period. Perhaps due to stress from repeated testing, FVB female mice actually had a net loss during this period (Fig. 4a, right panel). The accelerated body mass increase in cU2 mice is unlikely to have been due to increased food consumption; rather, it is more likely due to reduced energy expenditure. This is supported by the data showing normal daily food consumption, but with reduced body temperatures and physical activities in cU2 mice (Fig. S2).

**Figure 5:**
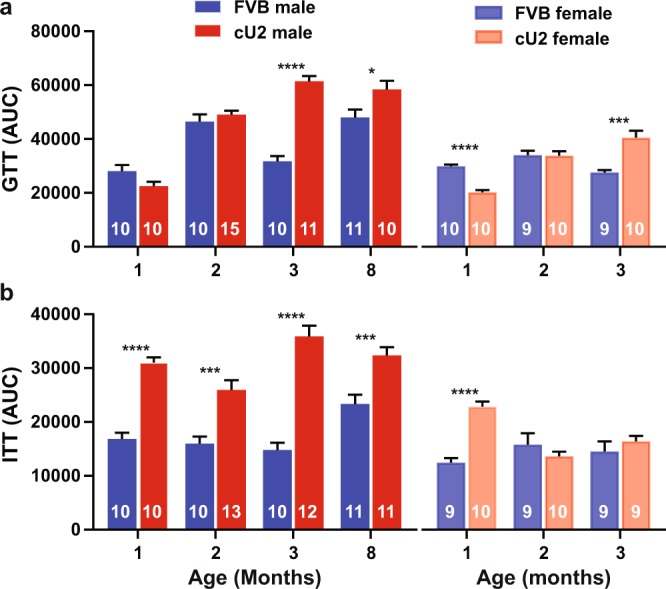
Glucose and insulin tolerance (GTT and ITT) assays. Changes in blood glucose levels were followed for up to 3 hours after glucose (**a**) or insulin (**b**) injection. Due to the multiple cohorts that vary in age, gender, and genotype, areas under the curve (AUCs) were used to simplify comparisons. Sample sizes are indicated within the bar graphs. Multiple *t*-tests were used to compare differences between age-matched FVB and cU2 mice. The false discovery rate was set at 1%. All data shown as mean ± SEM. *p < 0.05; **p < 0.01; *** p< 0.001; ****p < 0.0001.

Since cU2 mice had increased body mass and fat content, we wanted to evaluate glucose metabolism in both strains. Overall cU2 mice showed defects in glucose metabolism, displayed as slower glucose metabolism, and increased insulin resistance (Fig. 5). Despite the significant increase in fat content and body mass in cU2 mice by 2 months of age, both male and female cU2 mice showed relatively normal response in the GTT test up to this age (Fig. S3a,b). However, by 3 months of age, male and female cU2 mice showed significant delay in metabolizing glucose (Figs 5a and S3c,d). In contrast, male cU2 mice already showed a significant reduction in insulin sensitivity at one month of age (Fig. 5b), preceding the onset of significant increase in fat content and body mass (Fig. 4a,b). Furthermore, the extent of insulin resistance became progressively worse as the mice became older (Figs 5b and S4a,b). Female cU2 mice, on the other hand, showed variable results in insulin sensitivity across different age groups (Figs 5b and S4c,d). This inconsistency may be due to innate differences between different cohorts. Collectively, the data support an altered state of glucose metabolism in cU2 mice, with more severe symptoms observed in the male gender and increased insulin resistance as the earliest indicator.

### Altered adipogenic pathways in visceral fat tissue

**Figure 6:**
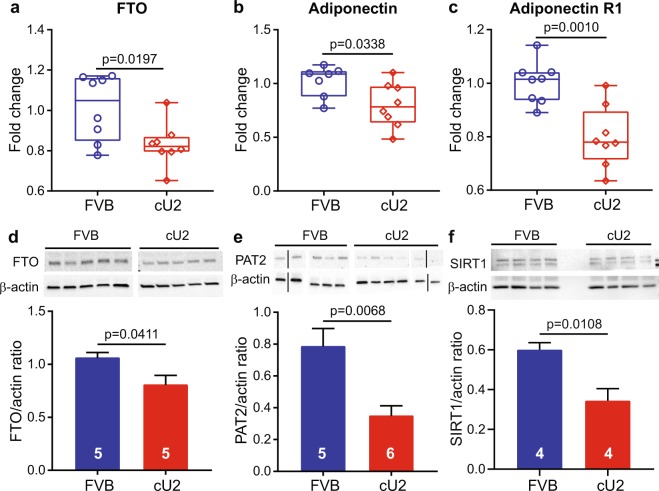
Obesity-associated gene and protein expressions in white adipose tissues at 3 months of age. (**a**–**c**) RT-qPCR assays for the expression levels of FTO (**a**); Adiponectin (**b**); and adiponectin R1 (**c**). (**d**–**f**) Western blot analysis of the protein levels of FTO (**d**); the brown/beige adipocyte marker PAT2 (**e**); and SIRT1 (**f**). Comparison of SIRT1 protein levels was performed on the full length SIRT1 protein (upper band). Cropped images of Western blot results are presented. Samples analyzed on separate blots are separated by a white space between the images; juxtaposed lanes that were non-adjacent in the gel are separated by a black line. Full-length blot images for FTO, PAT2, and SIRT1 are shown in Supplementary Information Fig. S6. Student’s *t*-test was used for the comparisons. Box plots (**a**–**c**) are used to show the relative gene expression levels between FVB and cU2, and Western blot data (d-f) are shown as mean ± SEM. Sample sizes for (**a**–**c**) are n = 8 each, and sample sizes for (**d**–**f**) are indicated within the bar graphs.

Increased body mass and fat composition and altered glucose metabolism in cU2 mice suggest underlying biochemical changes directing the trend towards dysregulation of adipogenesis. In this context, we selected 17 key genes involved in adipogenesis for RT-qPCR analysis of visceral fat tissues from 3-month-old male cU2 and control (FVB) mice (Figs 6 and S5). Among all the candidate genes, three showed significant down-regulation in cU2 visceral fat compared to age-matched controls; the three down-regulated genes were: *Fat Mass and Obesity Associated* (*FTO), Adiponectin*, and *Adiponectin receptor 1* (Fig. 6a–c). Since *FTO* is one of the essential genes associated with obesity signaling, we determined further that the decrease of FTO gene expression also corresponded to a reduction at the protein level (Fig. 6d). Further, we showed that the extent of browning in the white adipose tissue of cU2 mice was also significantly reduced, as indicated by the significant reduction of the mouse brown adipocyte marker – proton assistant amino acid transporter 2 (PAT2)^22^ (Fig. 6e). Consistent with increased adipogenesis and dysregulation of glucose metabolism in cU2 mice, protein levels of sirtuin 1 (SIRT1), a target gene of miR-34a and miR-34c and a known inhibitor of adipogenesis^23–25^, are significantly reduced in cU2 visceral fat (Fig. 6f). The molecular and biochemical data generated from cU2 mice suggesting increased adipogenesis and reduced browning were further supported by *in vitro* studies, in which, significantly higher yields of primary adipocytes and pre-adipocytes, as well as higher percentage of white adipocytes among these cells, were derived from visceral fat depots from cU2 mice (Fig. S7).

## Discussion

We report here the generation of a transgenic mouse line, cU2, with the human miR-34c transgene driven by a ubiquitin promoter for early and generalized expression^26^ of miR-34c in all tissues. The major findings are: insulin resistance occurring as early as one month of age, and significant gain in body mass and body fat composition at 2 months of age in both genders, with concomitant impairment of glucose metabolism and derailment of adipogenic regulation in visceral fat tissue. Collectively, our data suggest that ubiquitous overexpression of miR-34c may cause these early life physiological changes, which are recognized risk factors for adult obesity and diabetes, and that miR-34c transgenic mice may be a good animal model for unraveling the “Pandora’s box” of developmental origins of adult obesity.

Childhood and adolescent overweight originating from dysregulated physiological changes at the neonatal stage are often considered to be the underlying risk for obesity and metabolic syndrome in adulthood and old age^27^. Exposure to smoking, poor nutritional factors, and excessive maternal weight gain during pregnancy are among a few risk factors contributing to prenatal abnormalities and early life weight gain^28^. These risks may act alone or in concert to contribute deleterious stress at the genomic level, impacting perhaps most notably stress response molecular circuitry. The miR-34 cluster is the best-known noncoding RNA responder, *via* p53 activation with subsequent suppression of SIRT1 expression, a factor closely linked to adipogenesis^12,29–31^. We suggest that environmental exposure and nutritional choices can risk elevating expression of the miR-34 cluster in early life, leading to altered levels of adipogenesis and higher body weight. Among all the members of this microRNA cluster, miR-34c is abnormally regulated in both mouse adipocytes and human adipose tissue^16^. We thus generated the mouse model with overexpression of human miR-34c as our first attempt to determine the impact of this specific microRNA’s increase on the adipogenic pathway, as presented in this report.

Inter-animal and inter-gender variance are observed repeatedly in our results, such as the time window for decreased glucose metabolism and signs of insulin resistance (Figs 5, S3 and S4). Similarly, miR-34c levels spread from baseline to extremely high levels in the male liver samples examined (Fig. 2f). However, this fluctuation is less extreme in kidneys of the same animals (Fig. 2c), suggesting yet another level of variance, *i.e*. inter-tissue variance within the same animals. The variation of miR-34c and miR-34a expression levels is another example – whereas miR-34a levels are reduced in the kidney, and to a lesser extent in the liver, of male cU2 mice (Fig. 2b,e), they are upregulated in the adipose tissue of female cU2 mice (Fig. 3d). This inter-animal, -gender, and -tissue variance will only be explained by future larger animal cohort studies, which is beyond our current minimal design of ~10 animals per assay. Most importantly, tracking body weight and body fat gain by all the molecular and biochemical studies in individual mice of both genders, as well as behavioral studies, would be an ideal strategy to resolve inter-animal, -gender, and -tissue variation issues. Further work to determine the location and copy number of the transgene inserts in the mouse genome, and the interplay between the miR-34c transgene and the expression of endogenous members of this miRNA family, may shed light on inter-animal and -gender as well as inter-tissue variance. However, measurements of body fat composition and glucose metabolism show across-the-board significance of these two parameters, suggesting a positive trend of weight gain and associated abnormalities from hsa-miR-34c transgene expression.

The weight gain in cU2 mice differs from the popular high fat diet (HFD)-induced weight gain^32^, in that our transgenic mouse model gains weight without HFD or overeating. Both male and female cU2 mice manifest abdominal distension, along with increased body fat composition and abnormality in glucose metabolism, while on regular diet. Our results in 8-month-old male cU2 mice show that the transgenic mice do not eat more than the controls. However, the body temperature and physical activities in cU2 mice show a significant reduction from that of age-matched FVB controls. Dysregulation of several obesity-related signaling pathways, primarily the *FTO-IRX3* axis^33–35^ and *adiponectin/adiponectin R1*^36,37^, suggest that the presence of miR-34c transgenes leads to obesity-associated dysregulation of adipogenesis. In all, the weight gain and associated dysregulation of adipogenic pathways are most likely attributed to the impact of the human miR-34c transgene expression, rather than changes in expression of the endogenous miR-34 family. In particular, expression of the closely linked miR-34b, another member of the mouse miR-34b/c gene family^11^, does not show significant difference between cU2 and the non-transgenic controls (Fig. 3b,e). A recent publication shows that down regulation of miR-34a in high-fat diet-induced obese mice leads to a reduction in adiposity and promotes beige fat production in white adipose tissues by increasing mitochondrial energy metabolism and thermogenesis without reduction in food intake^13^. Given the overlapping biological functions between miR-34a and miR-34c, it is possible that miR-34c overexpression can lead to an opposite outcome with reductions in thermogenesis and energy metabolism and increase in adiposity in transgenic mice. Whether the reduced body temperature and physical activities in cU2 mice are due to reduced thermogenesis and mitochondrial energy metabolism at the cellular level, or due to changes in the control of energy homeostasis in the central nervous system will require careful investigation with cU2 mice at different ages in the future.

It is important to point out that of the two SIRT1 isoforms (*i.e*. full-length SIRT1 and exon 2- deleted SIRT1)^23^, only the full length SIRT1 level is reduced in the adipose tissue of cU2 mice. Since SIRT1 serves an inhibitory function for accumulation of white adipose tissue (WAT), its reduction in the cU2 visceral fat is not surprising. What is surprising, however, is that this reduction may not be due to the overexpression of miR-34c. Although all three miR-34 family members are highly homologous, recent large-scale gene array studies show only about 20% overlaps in their target genes in the metabolic signaling network between miR-34b/c and miR-34a^38^. This led us to suggest that the decrease of SIRT1 in cU2 visceral fat may not be due to the direct effect of miR-34c posttranslational suppression, but rather indirectly through a complex signaling network to regulate its activation and protein abundance^24,39–42^.

Currently, animal models of obesity are separated into two categories: mono- and poly-genic^1^. Mouse and rat models with mutations in the leptin pathway are the best representatives of the monogenic category^43–45^, while diet-induced models and several selectively bred mouse strains^46–48^ are frequently used models in the polygenic category. The monogenic models are seminal for unveiling the intricate networks governing leptin and its receptors, and their involvement in diabetes mellitus. On the other hand, the high-fat and/or high sucrose diets-induced obesity models are for simulating western style diets and the resulting metabolic disorders^49^. Regardless of the pathogenesis, all animal models show defects in the signaling networks controlling adipogenesis and insulin sensitivity. The cU2 mice with overexpression of human miR-34c may be considered as monogenic, due to the aberrant expression of one single gene. Functionally, however, it may be considered as polygenic because of the pleiotropic impact of miR-34c on multiple biochemical pathways. While well known for its tumor suppressing function, the miR-34 cluster is increasingly recognized for its impact on adiposity, insulin resistance signaling, and thermogenesis^13,50–52^. However, there has not been systematic examination, from birth to adulthood, of the cause-effect relationship between miR-34 and obesity in suitable animal models. While almost all established obesity models show increase in food consumption^1^, the cU2 mouse model is unique in that the weight gain and insulin resistance occur early in life without overeating and without high fat or high sucrose diet. Given the close connection between miR-34 and p53, and the role of p53 in sensing environmental stress, cU2 mouse model is also unique in that it provides a system to examine the connection between early life environmental exposure and the risk for adult obesity.

Like many other microRNAs, miR-34s are recently recognized as chief exosomal components secreted by adipocytes and resident adipose macrophages^53^, and significantly elevated levels of miR-34a have been shown in the plasma of patients newly diagnosed with type II diabetes^54^. There has been limited studies, but with increasing interests in recent years, to use circulating miRNAs as biomarkers to predict obesity in children^55^. Though it is not clear if overexpression of miR-34c and its sister miRNAs is related to early life obesity in human populations, this will be an important area of investigation in the future. Generating transgenic mice by overexpression of miR-34c is just a first step in opening Pandora’s box of molecular and environmental factors driving early childhood obesity. Much further work remains to be completed, including determining whether the same physiological changes occur with overexpression of sister microRNAs, miR-34a and miR-34b and how each member of the miR-34 cluster impact on the signaling pathways controlling glucose metabolism and adipogenesis^56–58^.

In conclusion, the transgenic mouse strain reported here, showing early life weight gain and visceral fat increase, appears to be an excellent model for childhood obesity. Although many questions remain yet to be answered, such as roles of other related miRNAs impacted by the persistent presence of hsa-miR-34c, our results suggest that overexpression of miR-34c may impact on adipogenic dysregulation and metabolic disorders in early life. Consequently, the mouse model may provide a window of opportunity for preventive intervention and diagnostics in children and young adults. One caveat of the mouse model presented here is that the transgene integration site has not been mapped. Given that transgene insertion in the host genome could inadvertently lead to transcriptional activation or interruption of endogenous gene(s) and that positional effect from the host genome on the transgene expression pattern could occur, these factors should be taken into consideration when interpreting the phenotype of cU2 mice.

## Methods

All animal-related studies adhered to guidelines in the Guide for the Care and Use of Laboratory Animals and were approved by the Institutional Animal Care and Use Committees (IACUCs) at the University of Louisville and the VA Palo Alto Health Care System. In both institutions, mice were housed in individually ventilated cages in a pathogen-free facility, with 12 hrs light/dark cycle and food and water provided *ad libitum*. The diet provided at the University of Louisville was the autoclavable Rodent Diet 5010 (LabDiet®, St. Louis, Missouri), and the diet provided at the VA Palo Alto Health Care System was the Teklad irradiated diet 2918 (Envigo, Indianapolis, Indiana). These two diets provide the same amount of metabolizable energy (3.1 kcal/g), with some differences in the composition of macronutrients (Table S1).

### Generation of transgenic mice

The cU2 transgenic mice used in this study were generated using a cassette composed of the human ubiquitin C promoter (hUbi), the *Escherichia coli LacZ* gene, the human miR-34c precursor gene, which was bracketed by the scaffold sequence of the human miR-33b gene, and finally the polyadenylation sequence (polyA) from the human growth hormone gene (Figs 1 and S1). The *LacZ* gene was used as a reporter of the transgene, and the human miR-33b scaffold sequence was used as a unique sequence for genotyping as well as qPCR validation of the transgene expression, since miR-33b is not present in the mouse genome. The entire cassette was inserted into the pPTuner plasmid (Clontech, Mountain View, California) for propagation.

Transgenic mouse generation was performed at the Cincinnati Children’s Hospital Medical Center (CCHMC) Transgenic Core, by pronuclear injection of fertilized eggs from FVB/N mice. Founders were identified by PCR genotyping, using primers within the human miR-33b scaffold sequence (Fig. S1). The founders were imported to the University of Louisville animal facility around 4 weeks of age, and male founders were crossed with female FVB/N mice (Harlan Laboratories, Indianapolis, Indiana) for germline transmission of the transgene. Hemizygous mating pairs were then used to generate homozygous cU2 in 4 generations of crosses. Homozygous cU2 mice were confirmed by test breeding to FVB/N mice to yield 100% transgenic offspring in >2 consecutive litters. Thereafter, homozygous intercrosses were used to generate all experimental animals. Age-matched FVB/N mice generated and maintained in the same housing facility were used as wild-type controls. Because the transgenic mice were generated in and backcrossed to FVB/N mice, they were considered congenic on the FVB/N background.

### Genotyping of transgenic mice

PCR genotyping for the transgene was performed using tail clippings taken from pups at 7–10 days of age. DNA was extracted using the Direct PCR lysis reagent (Viagen Biotech, Los Angeles, California), supplemented with Proteinase K (Qiagen, Hilden, Germany). PCR reactions were performed using the Phusion Hot Start II DNA Polymerase Master Mix (ThermoFisher, Carlsbad, California) with the forward (GGATTCAGCTTTCCATTCCCT) and reverse (AAGCAGGTCACACAGGAACAG) primers within the human miR-33b scaffold sequence (Fig. S1). PCR reactions included an initial cycle of 98 °C for 30 secs, followed by 35 cycles of 98 °C for 10 secs, 67.7 °C for 20 secs, and 72 °C for 30 secs, and a final cycle of 72 °C for 7 min. Transgenic mice were identified by the presence of a 475-bp band.

### Body composition measurement by dual energy X-ray absorptiometry (DEXA)

DEXA scan was used to measure the level of body fat and body mass as described^59^ in both male and female cU2 mice and age-matched FVB controls. A Discovery-A model DEXA scanner was adapted for rodent imaging (Hologic, Bedford, Massachusetts), and calibration was performed before each set of measurements. Mice were anesthetized with ketamine/xylazine cocktail for the procedure. Percent (%) of body fat data were taken from the region below the head, and body mass data were taken from global measurements. Due to the timing of mouse production, multiple cohorts of FVB and cU2 mice were used from 1 to 8 months of age. From ages 1 to 3 months, one group of male and female FVB mice were scanned monthly starting at one month of age, one group of male and female cU2 mice were scanned at one month of age, and a separate group of male and female cU2 were scanned at 2 and 3 months of age. To know whether body composition changes further as the mice age, separate groups of male FVB and cU2 mice were scanned at 8 months of age. The same mouse populations were also used for glucose tolerance and insulin tolerance tests (see below).

### Glucose tolerance and insulin tolerance tests

Glucose tolerance test (ipGTT) and insulin tolerance test (ipITT) were performed as described^60,61^. Mice were fasted for 16 and 4 hours for GTT and ITT, respectively, while water was supplied *ad libitum*. Baseline (time 0) blood glucose levels were measured before glucose or insulin injection. Glucose and insulin were then administered by intraperitoneal injection at 1 g/kg and 1 u/kg, respectively. Thereafter, blood glucose levels were monitored using a Contour blood glucose test meter and strips (Bayer, Boca Raton, Florida) at 15, 30, 60, 90, 120, and 180 minutes.

### RT-qPCR analyses of microRNAs and adipogenic genes

RNA was extracted from frozen liver, kidney, and adipose tissue collected from 3-month-old cU2 and FVB mice, using a miRNeasy kit (Qiagen, Hilden, Germany). Tissue pieces were first homogenized using a micro-grinder (RPI Corp, Mount Prospect, Illinois). Visceral fat homogenate was then spun in a microcentrifuge for 10 minutes at 12,000 × g. The resulting infranatant was collected and transferred to a clean microcentrifuge tube; the remaining lipid layer produced by adipose tissue was discarded. We followed Qiagen’s protocol for RNA isolation, and the remaining organic phase from each sample was stored at −80 °C for protein extraction. The concentration of the eluted RNA was measured on an Epoch spectrophotometer (Biotek, Winooski, Vermont), followed by quality control on an Agilent RNA 6000 Nano Kit chip (Agilent, Santa Clara, California). Samples with an RNA integrity number (RIN) below 9.0 were not included for further analysis.

For gene expression experiments, 100–500 nanograms of RNA were reverse transcribed into cDNA using a High-capacity RNA-to-cDNA™ kit (ThermoFisher, Waltham, Massachusetts) and an Applied Biosystems Veriti™ 96-well thermal cycler (ThermoFisher). Two to ten nanograms of cDNA were then applied to the qPCR reaction using the Taqman® Universal Master Mix II and the appropriate Taqman® 20X assay (ThermoFisher). The Taqman assays we used are presented in Table 1. The catalog number for all assays was 4331182.

**Table 1:** Taqman assays and assay IDs.

Gene	Assay ID	Gene	Assay ID	Gene	Assay ID
MEDAG	Mm00551008_m1	FTO	Mm00488755_m1	ADIPOR2	Mm01184032_m1
ADIPOR1	Mm01291334_mH	LEP	Mm01291334_mH	LEPR	Mm00440181_m1
ADIPOQ	Mm00456425_m1	CDH13	Mm00490584_m1	LEPROT	Mm00838516_g1
PPAR-γ	Mm00440940_m1	PPAR-α	Mm00440939_m1	RETN	Mm00445641_m1
IRX3	Mm00500463_m1	IRX5	Mm00502107_m1	INSR	Mm01211875_m1
18S rRNA	Mm04277571_s1	ESR1	Mm00433149_m1	ESR2	Mm00599821_m1

For miRNA quantification, reverse transcription was carried out using 10 nanograms of RNA and the Taqman® MiRNA Reverse Transcription Kit (ThermoFisher), along with the appropriate Taqman® MiRNA assay reagent. One microliter of cDNA was applied to each qPCR reaction along with Bullseye Taqprobe qPCR 2X Mastermix (MidSci, St. Louis, Missouri) and the appropriate Taqman® MiRNA assay reagent. The following assays, with assay IDs in parentheses, were used: miR-34a (000426) and miR-34c (000428); both probes detect both human and mouse miRNAs, due to highly conserved sequences of miR-34a and −34c between the two species. For miR-34b, mouse miR-34b (mmu-miR-34b) (002617) was used to detect the mouse-specific expression of this microRNA. Sno-202 (001232) was used as the internal control for consistent expression across different tissue types, and Cel-miR-54 (001361) as the spike-in control for cDNA synthesis.

Since human (hsa) and mouse (mmu) miR-34c sequences are 97.4% identical, to differentiate transgenic hsa-miR-34c expression from that of endogenous mmu-miR-34c, human miR-33b RT-qPCR assays were performed with specific probes to the miR-33b scaffold sequence (Fig. 1). Approximately 100–500 nanograms of RNA were applied to Bio-Rad’s (Hercules, California) iScript cDNA Synthesis Kit. Four microliters of cDNA were then added to each RT-qPCR reaction, along with Bullseye EvaGreen qPCR 2X Mastermix (MidSci), DMSO (6.5% final concentration), and human miR-33b primers. The primer sequences were: forward, TGTGGTAGGATCCCTTTGGA; and reverse, CTCTGGGAGGGGCAGGAT (Fig. S1). The melting temperature was set to 61 °C for 30 seconds.

All RT-qPCR experiments were performed in triplicate in either an Applied Biosystems® 7500 or Applied Biosystems® 7500 Fast instrument. For adipogenic gene expression experiments, 18S RNA was used as the reference gene; for microRNA experiments, sno-202 was used as the reference gene, and the spike-in Cel-54 was used as the control for cDNA synthesis. After normalization to their respective reference genes to obtain ∆Ct values, ΔΔCts between FVB and cU2 were calculated and converted to fold change values.

### Western blotting

Visceral fat protein was isolated from the organic phase produced by RNA extraction, as previously described^62^ for Western blot analysis. Briefly, visceral fat proteins were precipitated with 100% ethanol, spun, washed with 0.3 M guanidine hydrochloride in 95% ethanol, incubated in 100% ethanol, dried, and dissolved in RIPA buffer with added Protease/Phosphatase Inhibitor, using an RPI micro-grinder. Protein concentration was quantified using BCA assay (ThermoFisher). Twenty micrograms of visceral fat protein were separated by TGX Mini-protean gels (BioRad, Hercules, California), and transferred to PVDF membranes (ThermoFisher) for one hour at 100 volts. The membranes were blocked for one hour, then incubated overnight at 4 °C in the following primary antibodies: anti-PAT2 1:100 (sc-390969, Santa Cruz Biotechnology, Dallas, Texas); anti-SIRT1 1:100 (D1D7, #9475, Cell Signaling Technology, Danvers, Massachusetts), anti-FTO 1:500 (ab94482, Abcam, Cambridge, Massachusetts), and anti-β-actin 1:5000 (ab6276, Abcam). Blots were then incubated in either anti-mouse or anti-rabbit horseradish peroxidase (HRP)-conjugated secondary antibodies (1:8,000 or 10,000 respectively; Cell Signaling Technology) for one hour, developed with Supersignal West Pico chemiluminescent Substrate (ThermoFisher), and imaged on a ChemiDoc XRS + imaging system (BioRad). ImageJ software^63,64^ was used to quantify band intensities. The intensity of each target protein band was normalized to that of β-actin, to control for loading differences.

### Statistical analysis

All raw data were calculated initially using Microsoft Excel. Statistical software package Prism 7 (version 7.03, GraphPad Inc., La Jolla, California) was then used for comparisons across different genotypes, ages, and genders. Data collected from repeat measurements (GTT and ITT) were compared using two-way ANOVA with repeat measurements. Data collected from multiple time points (DEXA, GTT, ITT) were compared by multiple *t*-tests, with false discovery rate set at 1%. Discovery was determined using the two-stage linear step-up procedure of Benjamini, Krieger, and Yekutieli^65^. The computation assumed that all rows were sampled from populations with the same scatter. With the exception of RT-qPCR data, all data are presented as mean ± SEM. For all RT-qPCR experiments, box and whisker plots were used to graphically represent relative expression values. The box extends from the 25th to the 75th percentile, and the line in the middle of the box is plotted at the median. The whiskers then extend to the least and largest values in the dataset. Student’s *t-*test was used for single data point comparison between the two genotypes, and p-values < 0.05 were considered significant.

## Supplementary information

Supplementary Information

## Data Availability

All data generated or analyzed during this study are included in this published article and its Supplementary Information files.
