# Supplementary material for: Over-expression of miR-34c leads to early-life visceral fat accumulation and insulin resistance
Source: Sci Rep. 2019 Sep 25;9:13844. doi: 10.1038/s41598-019-50191-3 (PMC6761099; doi:10.1038/s41598-019-50191-3)
Supplement: Supplementary file 1 — Supplementary Information [file 41598_2019_50191_MOESM1_ESM.pdf]

SUPPLEMENTARY INFORMATION

Over-expression of miR-34c leads to early-life visceral fat accumulation and insulin resistance

Philip H. Jones[1], Brian Deng[2, 3], Jessica Winkler[1], Arin L Zirnheld[1], Sarah Ehringer[1], Vikranth Shetty[1], Matthew Cox[1], Huy Nguyen[3], Wen-Jun Shen[4, 5], Ting-Ting Huang[3, 5,*], and Eugenia Wang[1]

[1]Advanced Genomic Technology, LLC, Louisville, KY, USA; [2]Palo Alto Veterans Institute for Research, Palo Alto, CA, USA; [3]Departments of Neurology and Neurological Sciences and [4]Endocrinology, Stanford University School of Medicine, Stanford, CA, USA; [5]Geriatric Research, Education, and Care Center, VA Palo Alto Health Care System, Palo Alto, CA, USA

Corresponding author: Ting-Ting Huang
Department of Neurology and Neurological Sciences
Stanford University School of Medicine
VA Palo Alto Health Care System
Geriatric Research, Education, and Care Center
3801 Miranda Ave. Mail Stop 154I
Palo Alto, CA 94304
Tel. +1(650) 496-2581
E-mail: tthuang@stanford.edu

Figure S1

a Human miR-34c sequence and its flanking human miR-33b scaffold sequence

The human miR-34c sequence is underlined. The primers used for PCR genotyping of cU2 mice are indicated in bold letters, and the primers used for quantifying miR-34c transgene expression by RT-qPCR are marked with double underlines.

GTGAGGGGTGGGGTCAGGGGCCTGGCAGGGCTGGG**GGATTCAGCTTTCCATTCCCT**GGTTCCTCT
CCCCAGCCCCCAGGGGCTGCAGAAGACCATGGGGTTAGCCCAAGCAGCACAGGATAGGGGGTCCA
GCAGACCCTGCTTTTTGGCTAAGGCTTCTGTCCAGAGGAGAGGGGTTGCCCCTATCTGGCCTCAGTT
TCCCCATCCCTGGGAGGAGGGGGGTGGATGGTGTGGTAGGATCCCTTTGGAGGCCCTGCATCAGGA
GGGCTGGACAGCTGCTCCCGGGCCGGTGGCGGGTGTGGGGGCCGAGAGAGAGATCTCGAGAGTC
TAGTTACTAGGCAGTGTAGTTAGCTGATTGCTAATAGTACCAATCACTAACCACACGGCCAGGTAAAAA
GATTGAATTCTGCAGTCGACGGGCCCCCATCCTGCCCCTCCCAGAGCTGGAGCCCTGGTGACCCCT
GCCCTGCCTGCCACCCCCAGGCCGTGCAG**CTGTTCCTGTGTGACCTGCTT**GCATCCCTGTGACCCC
CTCCCCAGTGCCTCTCCTGGCCCTGGAAGTTGCCACTCCAGTGCCCACCAGCCTTG

Forward genotyping primer: **GGATTCAGCTTTCCATTCCCT**
Reverse genotyping primer: **AAGCAGGTCACACAGGAACAG** (complementary to actual sequence)

Forward RT-qPCR primer: TGTGGTAGGATCCCTTTGGA
Reverse RT-qPCR primer: CTCTGGGAGGGGCAGGAT (complementary to actual sequence)

Human miR-34c sequence:
AGTCTAGTTACTAGGCAGTGTAGTTAGCTGATTGCTAATAGTACCAATCACTAACCACACGGCCAGGTA
AAAAGATT

b Example of PCR genotyping results

Pups generated from test breeding of a homozygous cU2 (homozygous cU2 x FVB) mouse were genotyped using the PCR genotyping primers specified in above. DNA from the founder mouse was used as a positive control, and DNA from an FVB mouse was used as a negative control.

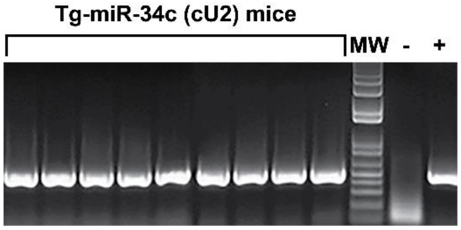

MW = molecular weight standard
- = Negative control (FVB DNA)
+ = Positive control (cU2 founder DNA)

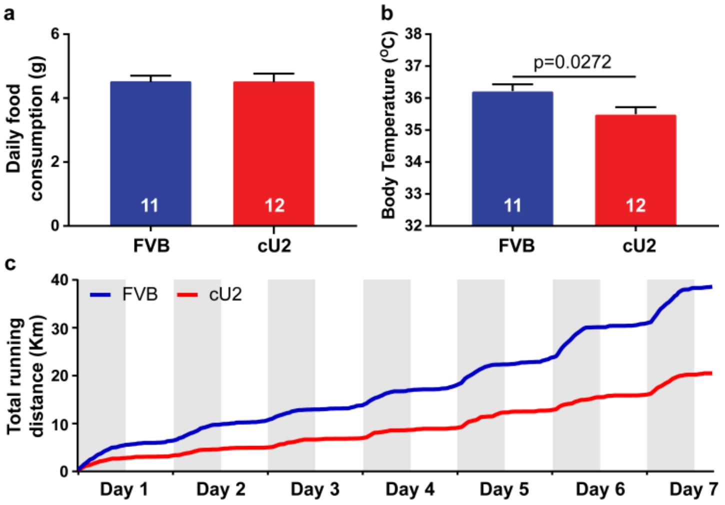

Figure S2 Daily food consumption and energy expenditure in cU2 mice. (a) Daily food intake is presented as the average of 4 daily measurements at 8 months of age. (b) Body temperatures, measured as rectal temperatures, were measured one week after completion of food intake study. (c) Voluntary wheel running was measured in 7 consecutive days and was used as an indicator of physical activities. Cumulative distances on the running wheel are presented. At the time of measurement, mice were between 5.5 and 6 months of age. The grey and white bars represent the dark and light phase, respectively. Cumulative activities in 30-min bins are plotted. Animal numbers for daily food consumption and body temperature measurements are indicated in the bar graphs; animal numbers for running wheel activities are n=8 for FVB and n=10 for cU2 mice. Data in (a) and (b) are shown as mean ± SEM; only group

averages are shown for each data point in (c). Student's t test was used for data analysis in (a) and (b); two-way repeat measure ANOVA was used for data analysis in (c). Although the average cumulative distance travelled in cU2 mice is only 52% of that in FVB controls, the difference between the two cohorts is not statistically significant due to large variations among different mice. However, there is a significant interaction between genotype and cumulative distance travelled ($F_{(321,5136)} = 2.048$, $p < 0.0001$). Detailed methods are described in Supplementary Methods at the end of this section.

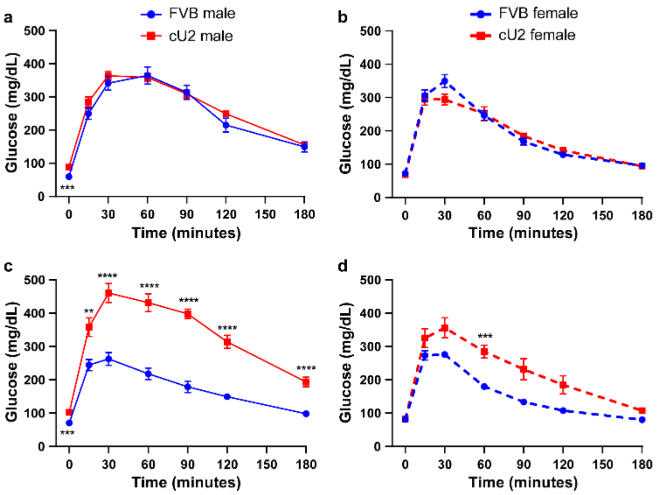

Figure S3 Blood glucose response curves from glucose tolerance tests. cU2 transgenic mice and age-matched FVB controls were subjected to glucose tolerance test at 2 months of age (a & b), and then again at 3 months of age (c & d). All data are presented as mean ± SEM. For some data points, the error bars are shorter than the height of the symbol and are not visible. Two-way repeat measurements ANOVA was used for comparisons at each time point. *, $p<0.05$; **, $p<0.01$; ***, $p<0.001$; ****, $p<0.0001$. N=10 for FVB males at 2 and 3 months; n=9 for FVB females at 2 and 3 months; n=15 and 11 for cU2 males at 2 and 3 months, respectively; n=10 for cU2 females at 2 and 3 months.

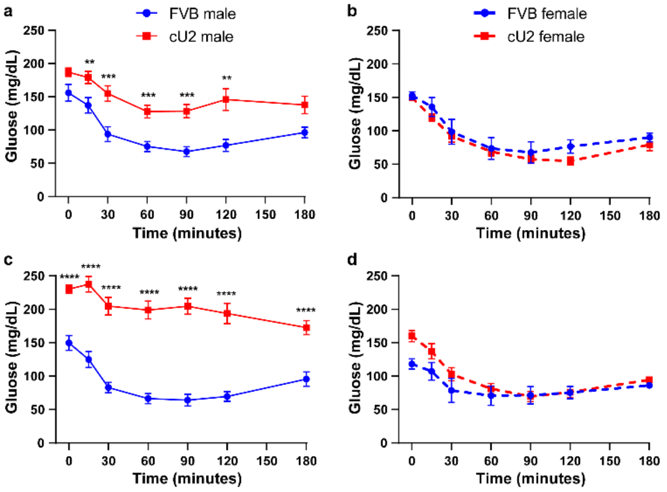

Figure S4 Blood glucose response curves from insulin tolerance test. cU2 transgenic mice and age-matched FVB controls were subjected to glucose tolerance test at 2 months of age (a & b), and then again at 3 months of age (c & d). All data are presented as mean ± SEM. For some data points, the error bars are shorter than the height of the symbol and are not visible. Two-way repeat measurements ANOVA was used for comparisons at each time point. **, $p<0.01$; ***, $p<0.001$; ****, $p<0.0001$. N=10 for FVB males at 2 and 3 months; n=9 for FVB females at 2 and 3 months; n=15 and 11 for cU2 males at 2 and 3 months, respectively; n=10 for cU2 females at 2 and 3 months.

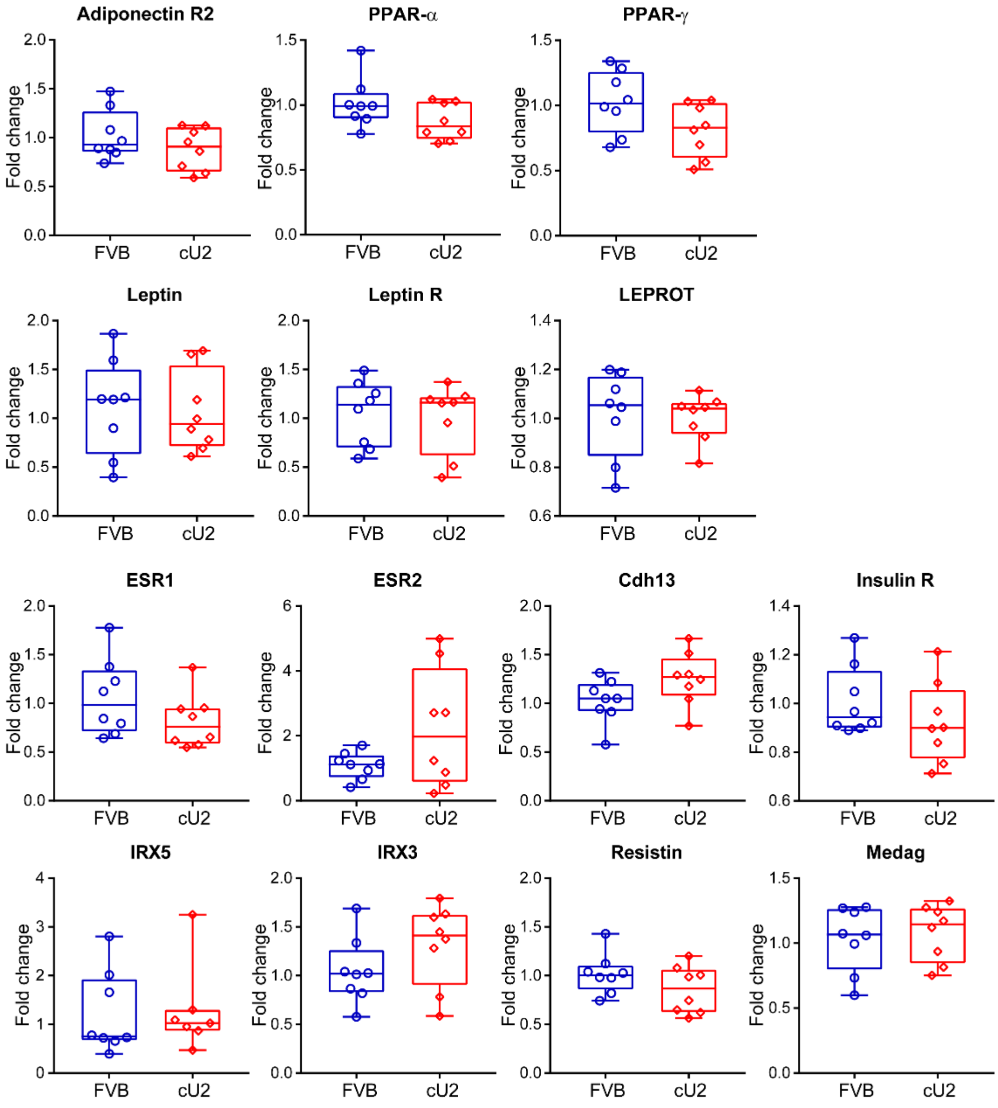

Figure S5 RT-qPCR quantification of obesity-associated genes in white adipose tissues at 3 months of age. N=7-8 each for final data analysis. Student's *t*-test was used for the comparisons. Box plots are used to show the relative gene expression levels between FVB and cU2.

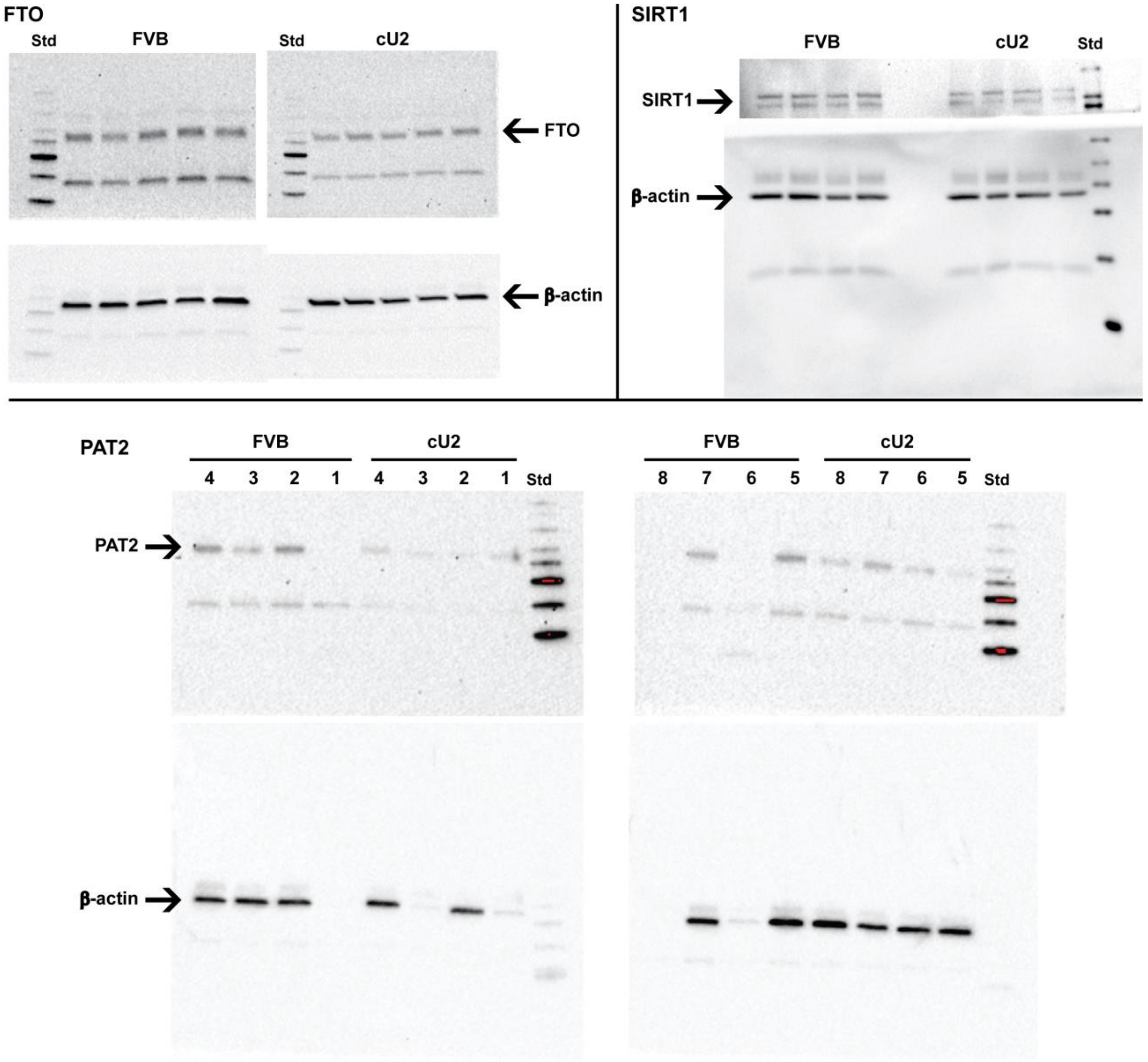

Figure S6 FTO, PAT2, and SIRT1 protein levels in white adipose tissues from 3 month old male FVB and cU2 mice. Full-length blot images for Western blot results presented in Figures 6 d-f. Std = MagicMark™ protein standards. All images were captured with the ChemiDoc™ XRS+ imaging system, and ImageJ was used to quantify band intensity of each protein. Adobe PhotoShop and Illustrator CC were used to crop and assemble the images for the presentations in Figures 6 d-f in the manuscript. For FTO, separate gels were used for the FVB and cU2 samples, but the blots were processed in parallel using identical conditions. Exposure time for FTO and β-actin was 358 seconds and 138 seconds, respectively. For PAT2, two blots were used to analyze 8 samples each from FVB and cU2 mice; the blots were processed in parallel using identical conditions. Loading and/or separation of some samples were not successful, as evidenced by the lack of β-actin signals. These samples (FVB: 1, 6, and 8; cU2: 1 and 3) were excluded from final quantification. Composite images of positive lanes from these two blots (FVB: 2-4, 5, and 7; cU2: 2, 4, 5-8) are presented in Figure 6e. Exposure time for PAT2 and β-actin was 600 seconds and 20 seconds, respectively. For SIRT1, a single gel was used for all samples from both strains. Due to the significant molecular weight difference between SIRT1 and β-actin, the blot was cut into two parts after transfer. The top portion was probed with anti-SIRT1 antibody, and the bottom part with anti-β-actin antibody. Exposure time for SIRT1 and β-actin was 240 seconds and 30 seconds, respectively.

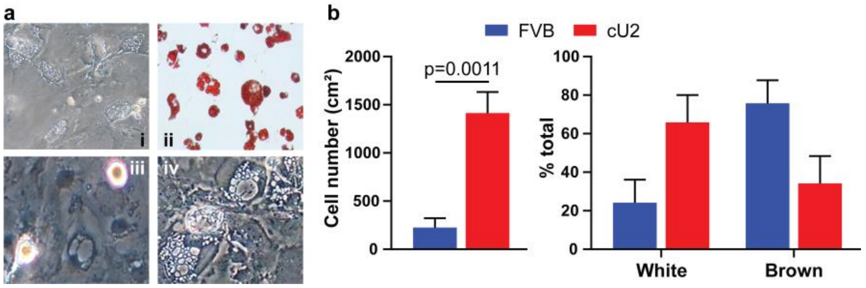

Figure S7 Quantification and differential count of primary adipocytes derived from visceral fat depot. (a) Images of primary adipocytes: i, phase contrast; ii, oil-red-O staining to highlight the lipid droplets in adipocytes; iii, a representative image of FVB adipocyte culture; iv, a representative image of cU2 adipocyte culture. (b) Adipocyte counts at 48 hours after plating and the differential count of white vs. brown adipocytes as a percentage of total adipocyte counts. Data are shown as mean ± SEM. N=5 each for the cell counts; n=6 each for the differential study. Cell counts were analyzed by Student's t test; differential counts were analyzed by two-way ANOVA corrected for multiple comparison (Sidak's method). There was a significant interaction between genotype and cell type ($F_{(1,20)} = 10.12$, $p = 0.0047$) in the differential count data. Detailed methods are described in Supplementary Methods at the end of this section.

Table S1 Comparison of autoclavable Rodent Diet 5010 and Teklad irradiated diet 2918

	Autoclavable Rodent Diet 5010[a]	Teklad irradiated diet 2918[b]
Metabolizable Energy (kcal/g)	3.08	3.1
Macronutrients		
Protein	24.6%	18.6%
Fat (ether extract)	4.8%	6.2%
Crude Fiber	4.1%	3.5%
Carbohydrates	50.1%	44.2%
Energy provided by		
Protein	28.7%	24%
Fat (ether extract)	12.7%	18%
Carbohydrates	58.5%	58%

Information extracted from documents from Lab Diet[a] (https://www.labdiet.com/cs/groups/lolweb/@labdiet/documents/web_content/mdrf/mdi4/~edisp/ducm04_028443.pdf) and Envigo[b] (https://www.envigo.com/resources/data-sheets/2018-datasheet-0915.pdf).

Table S2 Cycle threshold (Ct) values

Data from male mice from the original analysis

Ct	Adipose tissue				Kidney				Liver			
	sno202	miR-33b	miR-34a	miR-34c	sno202	miR-33b	miR-34a	miR-34c	sno202	miR-33b	miR-34a	miR-34c
FVB 1	15.17	33.07	18.02	22.11	17.31	10.77	19.67	24.76	16.27	11.43	19.67	23.96
FVB 2	15.30	22.39	18.74	22.93	17.40	10.70	19.38	24.77	14.10	10.00	18.09	22.77
FVB 3	15.11	17.15	19.40	23.43	17.18	10.21	19.48	25.07	16.08	11.69	21.82	24.74
FVB 4	14.24	30.64	18.09	21.92	17.11	9.98	19.53	24.58	16.31	11.23	19.76	23.41
FVB 5	15.34	24.60	20.65	24.00	17.09	9.97	19.73	24.75	15.25	12.32	20.73	25.26
FVB 6	15.74	35.51	19.75	24.15	17.41	10.37	19.09	24.18	16.32	10.81	19.13	24.11
FVB 7	15.60	25.76	21.43	24.83	17.68	10.00	19.48	24.05	16.80	10.60	19.02	24.06
FVB 8	15.00	26.30	19.76	24.54	17.26	10.72	19.28	24.48	17.26	17.42	19.94	24.79
FVB 9	15.31	37.75	21.56	23.99	16.98	10.60	19.91	24.28	15.42	11.16	20.15	24.40
cU2-1	15.35	16.28	19.75	18.22	17.08	10.11	21.91	22.86	15.94	15.13	24.92	26.06
cU2-2	15.45	16.36	20.49	20.04	17.65	9.98	20.97	22.09	16.69	9.11	22.00	23.66
cU2-3	15.07	16.40	19.07	18.78	17.35	9.64	20.78	22.64	16.05	10.33	21.31	23.05
cU2-4	16.38	16.23	17.55	17.74	17.45	9.67	21.65	22.64	15.50	8.44	21.02	21.77
cU2-5	14.90	17.64	18.70	18.69	17.35	9.40	21.38	22.00	16.99	7.74	21.07	22.06
cU2-6	15.94	25.50	20.65	19.42	17.38	10.15	21.59	22.80	14.38	8.39	21.72	23.71
cU2-7	15.40	21.66	18.29	19.72	17.45	9.86	21.77	22.53	16.75	8.48	19.88	22.52
cU2-8	15.20	23.80	20.99	21.52	17.15	9.62	21.52	21.95	15.50	12.30	21.94	26.03
cU2-9	13.70	23.68	18.87	21.49	17.26	9.56	21.25	21.95	15.93	6.57	19.27	18.67

Data from male and female mice -- viceral fat

Ct	Male viceral fat	
	Sno202	miR-34b
FVB 1	21.28	29.44
FVB 2	21.23	26.54
FVB 3	21.63	32.84
FVB 4	21.84	32.66
FVB 5	21.69	30.98
FVB 6	21.68	31.97
cU2-1	21.89	33.18
cU2-2	21.71	32.17
cU2-3	21.60	28.49
cU2-4	21.80	33.11
cU2-5	21.29	31.24
cU2-6	20.82	31.75

Ct	Female viceral fat			
	Sno202	miR-34a	miR-34b	miR-34c
FVB1	21.06	26.92	32.44	29.52
FVB2	20.53	25.86	31.86	29.09
FVB3	21.02	26.95	33.27	30.07
FVB4	20.28	25.43	31.39	29.55
cU2-1	20.75	25.05	31.29	29.25
cU2-2	20.87	25.38	33.67	30.53
cU2-3	21.12	24.45	31.43	30.11
cU2-4	21.91	24.76	30.89	29.55

Supplementary Methods

Food consumption. For daily food intake measurement, male mice were housed individually for the duration of the study. A sheet of filter paper was used to line the bottom of the mouse cage. Roughly 10 g of mouse chow was placed in the food tray each day. At the end of each 24-hour cycle, food crumbs on the filter paper were pooled with the remaining food. The weight difference in the mouse chow between the beginning and the end of the 24-hour cycle was recorded as food consumption. Cage and filter paper were replaced each day. Water was supplied *ad libitum*. Food intake was measured for 4 consecutive days, and the average was used for comparison between the two genotypes. Harland Teklad irradiated diet 2918 (Envigo, Indianapolis, Indiana) was used for the study.

Body temperature measurement. An Extech type K thermometer (model 421501) was used to measure rectal temperatures as specified by the manufacturer (Extech Instruments, Boston, MA) during the light phase. Mice were not anesthetized during the procedure.

Running wheel activity. To measure voluntary wheel running, 5.5-6 months old male FVB and cU2 mice were housed individually in Scurry Activity Wheel Chambers (Lafayette Instrument, Model 80821F) and given free access to running wheels for one week. Activities on the running wheels were monitored with Scurry Activity Monitoring software (Lafeyette Instrument, Model 86165); total running distance was recorded every 30 minutes. Data from a total of 160 hours of recording were used for the final analysis.

Primary adipocyte culture. Visceral fat tissues from 3-month old male cU2 and FVB controls were minced, then digested with 250 U/ml of collagenase II (Invitrogen, Carlsbad, California) at 37°C for 1 hour as described [1,2]. After removing undigested visceral fat, the stromal vascular cell fraction, which contained adipocytes and pre-adipocytes, was collected, washed with Dulbecco's phosphate buffered saline (DPBS), and plated in fibronectin-coated 24-well plates in Dulbecco minimal essential medium (DMEM) with 4.5 mg/ml glucose, 100 IU/ml Penicillin, and 100 µg/ml Streptomycin. Cells were incubated at 37°C with 5% CO2 for 48 hours to allow adipocytes to attach to the surface. Unattached cells were removed. The remaining attached cells were washed with PBS, and fresh DMEM with 4.5 mg/ml glucose and antibiotics was then added to the cells. The cell density at the initial plating was 1×10^5 (~70% confluence). Because the original cell pellet contains a mixed cell population, adipocytes are identified as cells with lipid droplets and confirmed with positive Oil-Red-O stain. Adipocytes in 5 random fields were counted at 200 x magnification.

To differentiate between brown and white adipocytes, primary adipocyte cultures were fixed with 4% paraformaldehyde, permeabilized 0.01% saponin and 2% BSA in DPBS, blocked with 5% BSA in DPBS, and then incubated in primary antibody to PAT2 [3] (Santa Cruz, sc390969; 1:200) in 5% BSA at 4°C overnight. Cells were then washed with PBS, and incubated for 1 hour at room temperature with Alexa-Fluor-488 goat-anti mouse (Invitrogen; 1:1000) secondary antibodies in 5% BSA. DAPI was used to stain the nuclei. Images were acquired using the Axiovert 200 inverted microscope; five random frames were used to count brown (labeled) and white (unlabeled) adipocytes with lipid droplets. In addition, nuclear position, either central (brown adipocytes) or peripheral (white adipocytes) can be localized by DAPI and was used as the second method to differentiate the two cell types.

References

1 Kang, J. H., Kim, C. S., Han, I. S., Kawada, T. & Yu, R. Capsaicin, a spicy component of hot peppers, modulates adipokine gene expression and protein release from obese-mouse adipose tissues and isolated adipocytes, and suppresses the inflammatory responses of adipose tissue macrophages. *FEBS Lett* **581**, 4389-4396, doi:10.1016/j.febslet.2007.07.082 (2007).

2 Hausman, D. B., Park, H. J. & Hausman, G. J. Isolation and culture of preadipocytes from rodent white adipose tissue. *Methods Mol Biol* **456**, 201-219, doi:10.1007/978-1-59745-245-8_15 (2008).

3 Ussar, S. *et al.* ASC-1, PAT2, and P2RX5 are cell surface markers for white, beige, and brown adipocytes. *Sci Transl Med* **6**, 247ra103, doi:10.1126/scitranslmed.3008490 (2014).
